# Multi-Omics Analysis of *Bombyx batryticatus* Formation Reveals Strain-Dependent Host Molecular Responses and Biomass Variation

**DOI:** 10.3390/jof12060398

**Published:** 2026-05-30

**Authors:** Qingqing Liu, Na Liu, Jia Fu, Yongting Bi, Yunqi Xie, Zhumei Jiang, Bin Chen, Shenghua Ying, Zhenghong Zhao, Yuejin Peng

**Affiliations:** 1 Yunnan State Key Laboratory of Conservation and Utilization of Biological Resources Kunming, College of Plant Protection, Yunnan Agricultural University, Kunming 650201, China; lq01067@163.com (Q.L.); 13769584395@163.com (N.L.); 17787989306@163.com (J.F.); kamibi825@163.com (Y.B.); 15288482002@163.com (Y.X.); jiangzhumei0520@163.com (Z.J.); chbins@163.com (B.C.); 2 State Key Laboratory for Vegetation Structure, Function and Construction (VegLab), College of Life Sciences, Zhejiang University, Hangzhou 310058, China; yingsh@zju.edu.cn; 3 Xinghui Agricultural Development Co., Ltd., Qvjing 655000, China; 15087447671@163.com

**Keywords:** *Bombyx batryticatus*, *Beauveria bassiana*, entomopathogenic fungi, transcriptomics, proteomics, immune response

## Abstract

*Bombyx batryticatus* is a traditional Chinese medicinal material derived from *Bombyx mori* infected by *Beauveria bassiana*; however, its formation mechanism remains poorly understood. This study compared infection processes in silkworms by two *B. bassiana* strains with markedly different virulence (highly virulent ZY027 and ARSEF2860). Integrated transcriptomic and proteomic analyses were employed to uncover, for the first time, the molecular basis of *B. batryticatus* formation at the systems biology level. The results demonstrated significant weight variations in *B. batryticatus* derived from different fungal strains. ZY027-induced stiff silkworms exhibited higher wet and dry weights than those infected by ARSEF2860. Large-scale gene reprogramming occurred in silkworm hemolymph post-infection, involving marked activation of Toll/Imd immune signaling pathways, ribosome biogenesis, and endoplasmic reticulum stress responses. A notable “uncoupling” between transcriptomic and proteomic profiles was identified, highlighting the critical role of post-translational regulation in host responses. The two strains triggered distinct metabolic reprogramming patterns: ZY027 notably suppressed oxidative phosphorylation and activated detoxification mechanisms, whereas ARSEF2860 presented characteristics of “immune–metabolic optimization.” These findings suggest that *B. batryticatus* formation involves complex fungus–silkworm molecular interactions in hemolymph, and that fungal strain characteristics are associated with significant differences in host molecular responses and product biomass. The study provides a theoretical foundation and innovative guidance for selecting strains with high *B. batryticatus* production potential and developing novel entomopathogenic fungal resources.

## 1. Introduction

*B. batryticatus* is a valuable traditional Chinese medicinal material, representing the dried and rigidified stage of silkworm (*Bombyx mori* Linnaeus) larvae. It is clinically used to treat stroke, strabismus, facial muscle twitching, and limb numbness, and occupies an important position in traditional Chinese medicine prescriptions [1]. In recent years, multiple bioactive components isolated from *B. batryticatus*, including proteins and peptides, fatty acids, flavonoids, nucleosides, steroids, coumarins, and polysaccharides, have demonstrated diverse pharmacological activities, such as anticonvulsant, anticoagulant, hypoglycemic, antitumor, anti-inflammatory, antioxidant, and immunomodulatory effects [2]. These findings provide scientific support for its traditional therapeutic applications [3,4].

The pathogen responsible for *B. batryticatus* formation is *Beauveria bassiana* (Bals.) Vuill., the key exogenous biological factor inducing silkworm rigidification. This fungus is a widely distributed entomopathogenic species with a broad host range [5]. Infection is initiated when conidia adhere to the insect cuticle. Under favorable conditions, conidia germinate to form germ tubes and appressoria, which penetrate the cuticle through mechanical pressure and the secretion of proteases and chitinases. The fungus then proliferates within the host, producing toxic secondary metabolites, including beauvericin, which ultimately cause host death and result in the characteristic “stiff silkworm” phenotype, marked by extensive mycelial growth and conidiation on both internal and external surfaces [6]. Therefore, *B. batryticatus* represents a distinct morphological manifestation of the complex pathophysiological processes occurring in *B. mori* following *B. bassiana* infection until death.

Modern research on *B. batryticatus* has primarily focused on the analysis of active components in the final product, isolation and identification of fungi from *B. batryticatus* (confirming *B. bassiana* as the primary infectious species [7,8,9]), and pharmacological studies [10,11]. Recent pharmacological research has classified the active ingredients into two main groups. The first group includes compounds with medicinal properties. For example, quercetin exhibits favorable binding interactions with RAC-alpha serine/threonine-protein kinase. Subsequent in vitro experiments confirmed the inhibitory effects of quercetin on PI3K and Akt phosphorylation levels [11]. Additionally, components such as nigrin, quercetin 3-O-neohesperidoside, and rutin have been identified to significantly improve nerve function damage [12,13]. The second group includes proteins extracted from *B. batryticatus*, showing potential anti-epileptic effects in vitro and possessing anti-inflammatory and neuroprotective activities. These proteins regulate the γ-aminobutyric acid (GABA) signaling pathway [14] and the NF-κB signaling pathway [15]. Furthermore, BB octapeptide, a novel peptide isolated from *B. batryticatus*, has been demonstrated as a highly effective inhibitor of platelet aggregation with low toxicity [16].

With the rapid advancement of omics technologies, powerful tools have become available for elucidating complex molecular regulatory networks within organisms at the systems biology level, especially useful for exploring complex dynamic systems such as host–pathogen interactions [17]. Transcriptomics can provide a comprehensive view of gene expression profile alterations in specific tissues or cells during biological processes (BPs), revealing key signaling pathways and regulatory factors [18]. Proteomics studies protein abundance, post-translational modifications, and interactions at the functional level, directly reflecting physiological states and BPs [19]. For example, comparative proteomic analysis revealed significantly decreased protein content in *B. batryticatus* processed by stir-frying with wheat bran, yet 32 proteins were identified as potential biomarkers [20]. However, a fundamental and unresolved questions remain: How exactly is *B. batryticatus* formed? Beyond physical colonization and growth by *B. bassiana*, how does this fungus influence and reshape the molecular-level life processes of *B. mori*? How does the host respond to this lethal invasion? This biological interaction is highly dynamic and complex. Current research lacks systematic and in-depth analyses of the host’s physiological response patterns, fungal infection strategies, and molecular interaction mechanisms between fungus and silkworm at the gene expression and protein network levels.

Our study found that *B. batryticatus* prepared using different *B. bassiana* strains displayed significant quality differences (weight). Taking silkworm hemolymph collected after *B. bassiana* infection as the research object, we integrated transcriptomic and proteomic analyses to systematically examine the molecular pathways associated with silkworm rigidification and death. Furthermore, we deeply explored the molecular basis underlying fungus–silkworm interactions. This study investigates the molecular responses in silkworm hemolymph during *B. batryticatus* formation using two *B. bassiana* strains with different virulence. By integrating transcriptomic and proteomic analyses of hemolymph at a single infection time point, we aim to characterize strain-dependent host molecular reprogramming patterns. While wet and dry weights were used as initial indicators of production yield, we acknowledge that these measurements do not fully represent medicinal or pharmacognostic quality. This work provides a foundation for understanding fungus–silkworm interactions in hemolymph and identifies candidate pathways for future quality-related investigations.

## 2. Materials and Methods

### 2.1. Fungi and Culture Media

Microbial strains were cultivated according to previously described methods [21]. The wild-type *B. bassiana* strain ARSEF2860 was obtained from the USDA Agricultural Research Service Collection of Entomopathogenic Fungal Cultures. The ZY027 strain was isolated in 2025 from infected silkworms in Gejiu City, Honghe Hani, and Yi Autonomous Prefecture, Yunnan Province, China. Wild-type strains and their derivatives were cultured on Sabouraud dextrose agar (SDAY: 4% glucose, 1% peptone, 1.5% agar, and 1% yeast extract) at 25 °C under a 12 L:12 D photoperiod for 7 days. Colony diameters were subsequently measured and photographed. Each strain was tested with three replicates, accompanied by three parallel controls per experiment.

The isolated *B. bassiana* strain ZY027 underwent molecular identification using ITS1 paired primers (CTTGGTCATTT/AGAGGAAGTAA) for amplification. ITS1 sequences from closely related strains were obtained through the NCBI database. Phylogenetic relationships among ITS genes from other *B. bassiana* strains were analyzed using the neighbor-joining method in MEGA7 software (http://www.megasoftware.net), and a phylogenetic tree was constructed [22].

### 2.2. Silkworm Rearing

Silkworms were provided by Xinghui Agricultural Development Co., Ltd., Luliang County, Qujing City, Yunnan Province, China. The insects were maintained in rooms at 25 °C and approximately 70% humidity. Fresh mulberry leaves were harvested daily, washed, drained, and then used for feeding. Silkworm larvae at 1st–2nd, 3rd–4th, and 5th instar stages were housed separately. For larvae at the 1st–2nd instar, mulberry leaves were cut into approximately 5 mm-wide pieces before feeding.

### 2.3. Fungal Virulence Determination and B. batryticatus Quality Assessment

Approximately 150 healthy fifth-instar silkworms (6–8 days old) were selected and divided into three groups, each containing 50 silkworms. Conidial suspensions (1 × 10^8^/mL) of *B. bassiana* strains ZY027 and ARSEF2860 were evenly sprayed onto the body surfaces of the silkworms. Silkworms were placed in rearing trays, ensuring proper ventilation and avoiding stacking, to facilitate synchronous infection. Silkworm mortality was recorded every 24 h from day 4 to day 6 post-treatment. Subsequently, dead silkworms were transferred to ventilated gauze paper, and each silkworm cadaver was weighed (wet weight). Approximately 20 cadavers per replicate were measured. Cadavers were then air-dried naturally at 25 °C for 5 days, after which dry weight was measured individually, again using approximately 20 cadavers per replicate. The experiment was repeated three times with three parallel controls.

### 2.4. Transcriptome and Proteome Sample Preparation

After culturing *B. bassiana* strains for 7 days, mature conidia were harvested and suspended in sterile water to a concentration of 1 × 10^8^ conidia/mL. Healthy silkworm larvae were infected by spraying. A 0.02% Tween 80 solution served as the control group [23]. Treated silkworms were placed in rearing trays at 25 °C for 3 days. The abdominal legs of silkworms were cut open using sterile scissors, and 100 μL hemolymph was collected using a pipette. Hemolymph samples were mixed at a 1:3 (V:V) ratio with anticoagulant (62 mM NaCl, 100 mM glucose, 30 mM sodium citrate, 26 mM citric acid, 20 mM EDTA, pH 4.6), snap-frozen in liquid nitrogen, and stored at −80 °C before transcriptome and proteome sequencing by Biomarker Technologies Co., Ltd. The samples for RNA-seq analysis included three replicates of *B. batryticatus*, while the samples for label-free proteomic analysis included five replicates.

### 2.5. Transcriptomic Analysis

Sequencing was conducted on the Illumina Novaseq platform, following previous procedures [24]. Clean reads were obtained by removing low-quality reads and those containing adapters or poly-N sequences. Reference genome and gene annotation files were retrieved from the NCBI database (https://www.ncbi.nlm.nih.gov/). RNA-seq libraries were sequenced on the Illumina NovaSeq 6000 platform (Biomarker Technologies Co., Ltd., Beijing, China) with paired-end 150 bp reads. Raw sequencing data were deposited in the Genome Sequence Archive (GSA) under accession number CRA039075. Clean reads were obtained by removing adapter sequences, reads containing >10% ambiguous bases (N), and low-quality reads (Q < 20). The reference genome *B. mori* (ASM15162v1, GCF_000151625.1) and gene annotation files were retrieved from NCBI. Hisat2 v2.0.5 was used to build the genome index, and clean reads were aligned with default parameters. Mapping rates ranged from 85.3% to 91.7% across samples. FeatureCounts v1.5.0-p3 was used to count reads mapped to genes. Three biological replicates were included for each treatment group (Control, ZY027, ARSEF2860). Fragments per kilobase of transcript per million mapped fragments (FPKM) were calculated based on gene length and mapped reads. Differentially expressed genes (DEGs) were identified using DESeq with criteria of an adjusted *p*-value ≤ 0.05 and |log_2_Fold change| > 1. Gene Ontology (GO) and Kyoto Encyclopedia of Genes and Genomes (KEGG) enrichment analyses of DEGs were performed with the ClusterProfiler R package. Additionally, protein–protein interaction networks, Gene Set Enrichment Analysis (GSEA), differential gene co-expression analysis, and gene co-expression network construction (WGCNA) were conducted.

### 2.6. Proteomic Analysis

The samples were processed as previously described [24]. Protein solutions were reduced with 10 mM DTT at 56 °C for 1 h, then cooled to room temperature. Subsequently, 55 mM IAM was added for alkylation in the dark for 45 min. Desalting was performed using ultrafiltration (3 kDa) or acetone precipitation, followed by lyophilization at −80 °C. The samples were sent to Biomarker Technologies Co., Ltd. (Beijing, China) for proteome sequencing and analyzed via the BMKCloud platform (www.biocloud.net) at Biomarker Technologies. Each library was replicated independently three times. All acquired data were mapped to the *B. mori* proteome database. Differentially expressed proteins (DEPs) between the two libraries were identified using the Cuffdiff method, applying a threshold of q-value < 0.05 (false discovery rate of 5%) and absolute log_2_ (fold-change) > 1. DEP enrichment analysis was conducted using the online FungiFun2 tool (https://elbe.hki-jena.de/fungifun/) (21 February 2026), applying a corrected *p*-value threshold of <0.05.

### 2.7. Transcriptome and Proteome Joint Analysis

As previously described [24], joint analyses of proteomic and transcriptomic data identified DEGs and DEPs. The results underwent nine-quadrant analysis and KEGG enrichment analysis. Pearson correlation coefficients between DEPs and corresponding mRNA expression levels were calculated.

### 2.8. Statistical Analysis

Data meeting assumptions of normality and homogeneity of variance were analyzed using unpaired *t*-tests. For multiple-group comparisons, one-way and two-way ANOVA followed by Tukey’s honest significance difference (HSD) tests were applied. Statistical significance was defined as *p*-value < 0.05.

## 3. Results

### 3.1. Significant Weight Differences in B. batryticatus Prepared by B. bassiana Strains ARSEF2860 and ZY027

**Figure 1:**
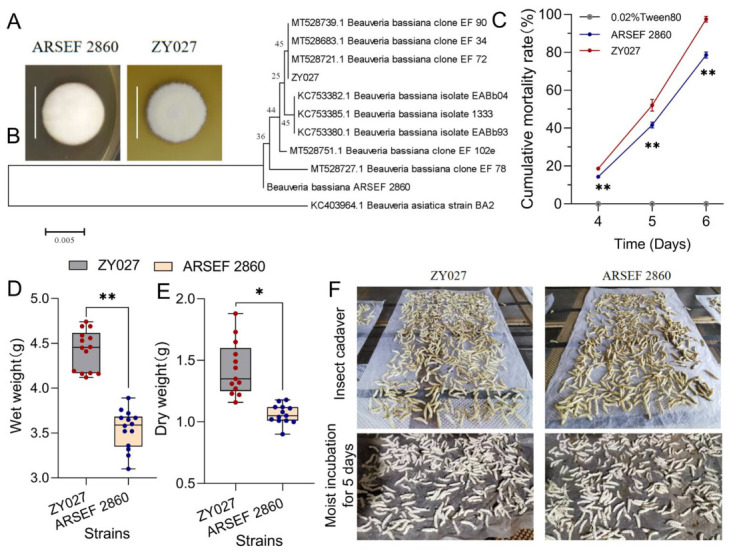
Comparison of *B. batryticatus* preparation using *B. bassiana* strains ZY027 and ARSEF2860. (**A**) Colony morphology of *B. bassiana* strains ZY027 and ARSEF2860 on SDAY plates at day 7. (**B**) Molecular identification of the two fungal strains through phylogenetic tree constructed using neighbor-joining method. (**C**) Cumulative mortality of 5th instar silkworms infected by *B. bassiana* strains ZY027 and ARSEF2860. (**D**,**E**) Biomass comparison of silkworms infected by the two *B. bassiana* strains, including wet weight (**D**) and dry weight (**E**). (**F**) Morphology of *B. batryticatus* prepared from *B. bassiana* strains ZY027 and ARSEF2860, showing insect cadavers and finished products. Tukey′s honestly significant difference [HSD]: *p* < 0.05 (*), *p* < 0.01 (**); error bars: standard deviation.

Morphological identification indicated that ZY027 and ARSEF2860 strains exhibited similar morphology on SDAY plates at day 7 (Figure 1A). Molecular identification confirmed that the ZY027 strain belongs to *B. bassiana* (Figure 1B). Bioassays with fifth-instar silkworms revealed that mortality caused by ZY027 was significantly higher than by ARSEF2860. Mortality rates reached 50% and 40% on day 5 for ZY027 and ARSEF2860, respectively (Figure 1C). Both wet weights (Figure 1D) and dry weights (Figure 1E) of *B. batryticatus* formed after infection by ZY027 were significantly higher than those infected by ARSEF2860.

### 3.2. Transcriptomic Analysis Reveals Differential Gene Expression in Silkworm Hemolymph Influenced by Fungal Strain

**Figure 2:**
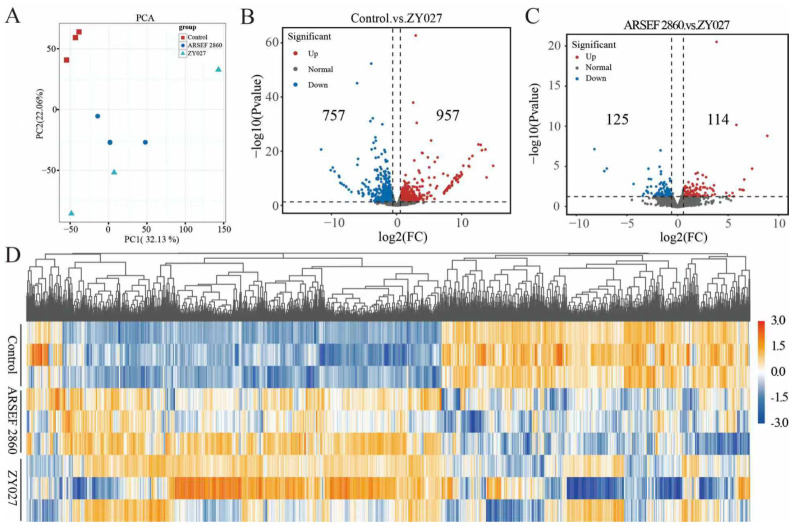
Comparative transcriptomic analysis of silkworm hemolymph infected by *B. bassiana*. (**A**) PCA analysis of hemolymph samples from healthy (control) silkworms and those treated with *B. bassiana* ARSEF 2860 and ZY027 strains. (**B**,**C**) Volcano plots of differentially expressed genes in hemolymph from healthy silkworms and those treated with *B. bassiana* ARSEF 2860 and ZY027 strains, including control vs. ZY027 (**B**) and ARSEF 2860 vs. ZY027 (**C**). (**D**) Heatmap of differentially expressed genes in hemolymph from healthy silkworms and those treated with *B. bassiana* ARSEF 2860 and ZY027 strains.

Principal component analysis results indicated no evident outlier samples. Replicates within each treatment group clustered closely together, confirming the reliability and reproducibility of the experiment (Figure 2A). Differential expression analysis identified 1714 significant DEGs between control and ZY027-treated groups, comprising 957 up-regulated genes (red dots) and 757 down-regulated genes (blue dots) (Figure 2B). Between ZY027 and ARSEF2860 strain treatments, 239 significant DEGs were identified, including 114 up-regulated and 125 down-regulated genes (Figure 2C). Heatmap clustering clearly distinguished healthy silkworm groups from fungus-infected silkworm hemolymph samples (Figure 2D).

**Figure 3:**
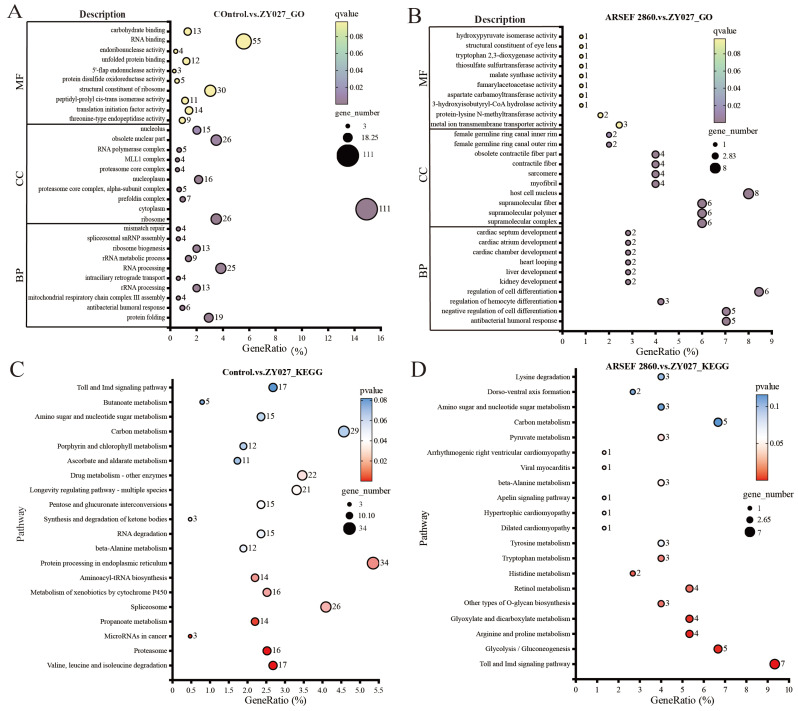
Enrichment analysis of differentially expressed genes in silkworm hemolymph identified through comparative transcriptomics. (**A**,**B**) GO enrichment analysis of differentially expressed genes in silkworm hemolymph for control vs. ZY027 (**A**) and ARSEF 2860 vs. ZY027 (**B**). Top 10 terms per ontology (BP/CC/MF) for up-regulated and down-regulated genes. (**C**,**D**) Top 20 pathways ranked by significance in KEGG enrichment analysis of differentially expressed proteins in silkworm hemolymph for control vs. ZY027 (**C**) and ARSEF 2860 vs. ZY027 (**D**).

To elucidate the functional implications of these gene expression differences, GO enrichment analysis was performed separately on up-regulated and down-regulated gene sets. Significantly enriched GO terms emerged across all three categories: BP, Cellular Component (CC), and Molecular Function (MF). Between control and ZY027 strain groups, significant functional reprogramming was observed across all three GO categories (Figure 3A). In the MF category, enriched terms included RNA binding and endoribonuclease activity, indicating roles in glycan-recognition-based immune surveillance and RNA processing during pathogen challenges. Notably, structural constituents of ribosomes emerged as prominent terms, highlighting extensive remodeling of translation machinery. The CC category showed significant enrichment of extracellular region, membrane, and cytoplasm terms, reflecting the active secretion of immune effectors into hemolymph. The prominent enrichment of extracellular components underscored characteristic humoral immune responses in the insect open circulatory system. In the BP category, enriched terms such as small ribosomal subunit assembly, rRNA metabolic processes, and rRNA processing collectively indicated active rRNA biosynthesis. Additionally, enrichment of protein folding and antimicrobial humoral immune response terms suggests coordinated efforts in maintaining protein homeostasis and innate immune activation.

KEGG pathway enrichment analysis revealed that the Toll and Imd signaling pathways were the most significantly enriched, confirming the activation of the insect innate immune mechanism. Meanwhile, the enrichment of butanoate metabolism and amino sugar and nucleotide sugar metabolism indicated energy redistribution toward defensive glycosylation processes. Additionally, significant enrichment of carbon metabolism and pyruvate metabolism suggested reprogramming of central carbon metabolism. Notably, the activation of the longevity-regulating pathway implied stress-induced aging and transcriptional quality control mechanisms. Enrichment of protein processing in the endoplasmic reticulum pathway indicated the activation of the endoplasmic reticulum stress response and unfolded protein response (UPR). Enrichment of spliceosome and RNA polymerase further supported the enhancement of transcriptional and post-transcriptional regulation under infection. Pathways related to drug metabolism-cytochrome P450 and metabolism of xenobiotics by cytochrome P450 reflected detoxification responses to pathogen metabolites or host-derived damage-associated molecular patterns (Figure 3B).

In contrast, the GO enrichment profiles for the ZY027 and ARSEF2860 strain treatment comparison (Figure 3C) showed significantly attenuated characteristics, suggesting that the biocontrol agent exerts a protective regulatory effect on transcriptional responses. In the MF category, enrichment of RNA binding and ribosomal structural constituents was notably reduced. Instead, terms such as hydroxypyruvate isomerase activity and tryptophan 2,3-dioxygenase activity became predominant, indicating a shift toward metabolic pathway optimization rather than emergency ribosomal synthesis. CC enrichment highlighted contractile fibers and supramolecular fibers as key terms. The BP category prominently featured regulation of cell differentiation along with a sustained antimicrobial humoral response, suggesting developmental stability induced by ARSEF2860 alongside controlled immune activity, in contrast to the strong stress response observed in Figure 3B.

KEGG analysis (Figure 3D) showed pathway structures differed significantly from direct infection scenarios. The Toll and Imd signaling pathways remained enriched but involved fewer genes, indicating preserved yet attenuated immune signaling. Increased representation of genes associated with glycolysis/gluconeogenesis and carbon metabolism indicated active optimization of metabolic pathways. Persistent enrichment of amino sugar and nucleotide sugar metabolism suggested maintained glycosylation capability essential for immune recognition. Distinct enrichment of glyoxylate and dicarboxylate metabolism and arginine and proline metabolism suggested metabolic diversification driven by ARSEF2860 to enhance energy efficiency. Enriched pathways of tyrosine and tryptophan metabolism implied aromatic amino acid catabolism for neurotransmitter or melanin synthesis, potentially facilitating immune activation and wound healing. Compared to Figure 3C, the reduced enrichment of pathways such as RNA degradation, spliceosome, and protein processing in the endoplasmic reticulum indicated alleviation of transcriptomic stress and restoration of protein homeostasis, reflecting adaptive protection rather than emergency damage control.

### 3.3. Proteomics Reveals Differential Protein Expression in Silkworm Hemolymph Influenced by Insect Health Status

**Figure 4:**
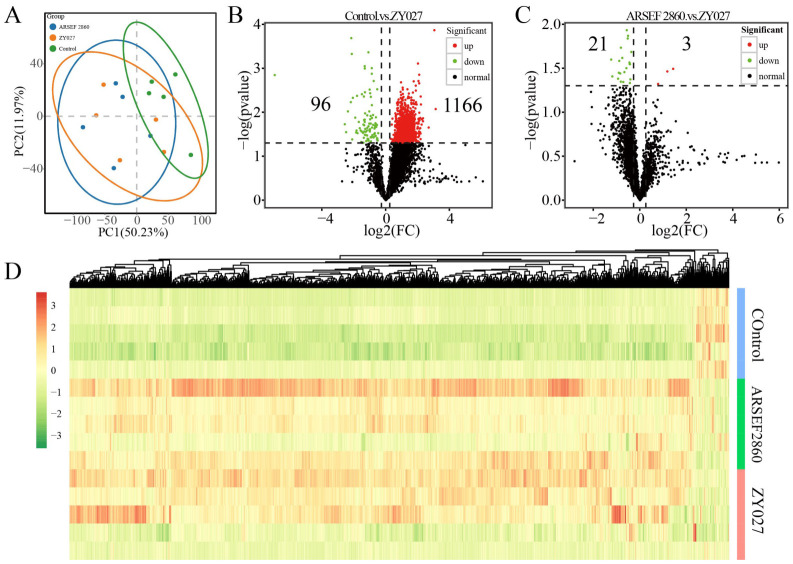
Proteomic analysis of silkworm hemolymph infected by *B. bassiana*. (**A**) PCA analysis of hemolymph samples from healthy (control) silkworms and those treated with *B. bassiana* strains ARSEF 2860 and ZY027. (**B**,**C**) Volcano plots of differentially expressed proteins in hemolymph from healthy silkworms and those treated with *B. bassiana* strains ARSEF 2860 and ZY027, including control vs. ZY027 (**B**) and ARSEF 2860 vs. ZY027 (**C**). (**D**) Heatmap of differentially expressed proteins in hemolymph from healthy silkworms and those treated with *B. bassiana* strains ARSEF 2860 and ZY027.

Principal component analysis results indicated no obvious outliers (Figure 4A). Quantitative proteomic analysis identified 1362 proteins with significant differential abundance (DEPs) between control and ZY027-treated groups, including 96 up-regulated and 1166 down-regulated proteins (Figure 4B). Between ZY027 and ARSEF2860 treatment groups, 24 proteins showed significant differential abundance, comprising three up-regulated and 21 down-regulated proteins (Figure 4C). Volcano plots revealed a smaller dynamic range of protein abundance changes compared to transcriptomic analysis, suggesting strong regulation at the transcriptional level. Hierarchical clustering of DEPs demonstrated clear distinctions in protein expression signatures between healthy and *B. bassiana*-infected silkworms (Figure 4D).

**Figure 5:**
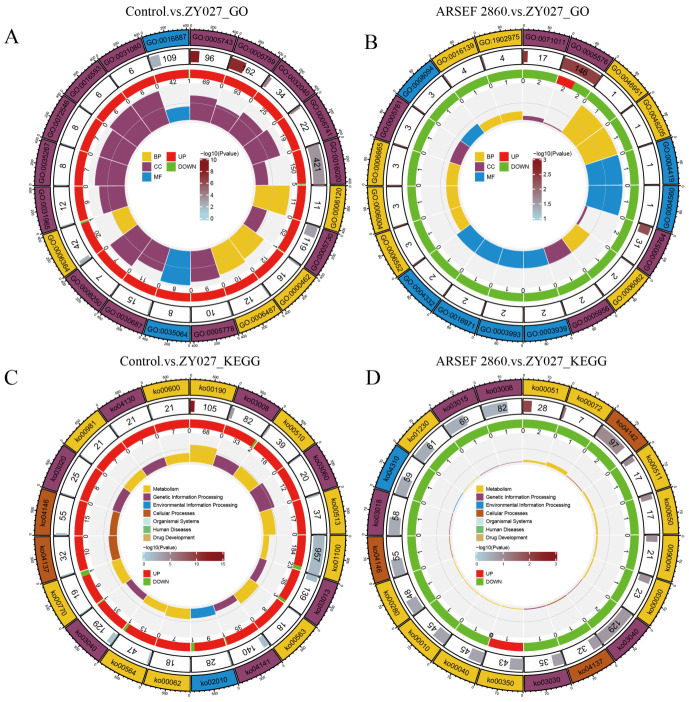
Enrichment analysis of differentially expressed proteins in silkworm hemolymph screened through proteomics. (**A**,**B**) GO enrichment analysis of differentially expressed proteins in silkworm hemolymph from control vs. ZY027 (**A**) and ARSEF 2860 vs. ZY027 (**B**) comparisons. (**C**,**D**) KEGG enrichment analysis of differentially expressed proteins in silkworm hemolymph from control vs. ZY027 (**C**) and ARSEF 2860 vs. ZY027 (**D**) comparisons.

GO enrichment analysis indicated distinct functional domain distributions of DEPs between control and ZY027-treated groups (Figure 5A), including ATP hydrolysis activity/ATPase activity (GO:0016887), methylated histone binding (GO:0035064), rRNA processing (GO:0006364), endoplasmic reticulum membrane (GO:0005789), and membrane (GO:0016020). For ZY027 versus ARSEF2860 treatments, enriched functional domains included glucosinolate biosynthetic process (GO:0016139), mitotic DNA replication initiation (GO:1902975), extracellular region (GO:0005576), lysosome (GO:0005764), and DNA helicase activity (GO:0008094) (Figure 5B).

KEGG pathway analysis revealed that, relative to the control, 20 pathways exhibited significant dysregulation in hemolymph from ZY027-treated silkworms. These pathways included metabolic pathways (ko01100), oxidative phosphorylation (ko00190), RNA transport (ko03013), protein processing in endoplasmic reticulum (ko04141), spliceosome (ko03040), and ribosome biogenesis in eukaryotes (ko03008) (Figure 5C). Metabolic pathways were the most represented, involving 957 up- or down-regulated proteins. Compared to ARSEF2860-treated silkworms, ZY027 treatment significantly dysregulated 20 pathways, prominently involving spliceosome (ko03040), lysosome (ko04142), mRNA surveillance pathway (ko03015), ribosome biogenesis in eukaryotes (ko03008), Wnt signaling pathway (ko04310), RNA degradation (ko03018), and peroxisome (ko04146) (Figure 5D). The spliceosome pathway ranked highest, covering 129 up- or down-regulated proteins.

### 3.4. Joint Transcriptomic and Proteomic Analysis Reveals Strain-Dependent Host Molecular Responses in Hemolymph

**Figure 6:**
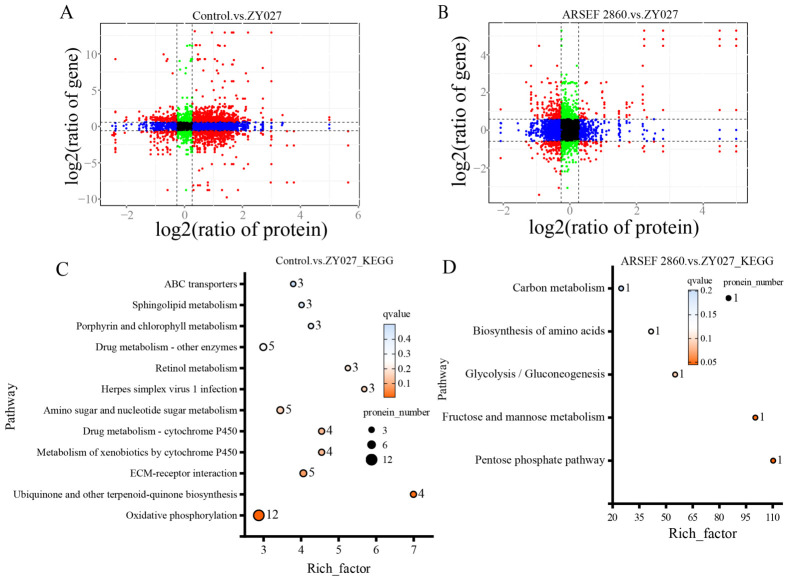
Integrated transcriptomic and proteomic analysis of silkworm hemolymph. (**A**,**B**) Nine-quadrant analysis showing correlations between differentially expressed genes (DEGs) and differentially expressed proteins (DEPs) in hemolymph from healthy (control), ARSEF 2860- and ZY027-treated silkworms, including control vs. ZY027 (**A**) and ARSEF 2860 vs. ZY027 (**B**). (**C**,**D**) KEGG enrichment analysis of DEPs identified through integrated analysis, including control vs. ZY027 (**C**) and ARSEF 2860 vs. ZY027 (**D**).

Nine-quadrant analysis comparing control and ZY027 groups showed significant divergence between transcriptomic and proteomic changes (Figure 6A). The analysis of 4433 gene–protein pairs revealed a moderate overall correlation but notable dispersion around the diagonal. Consistent regulation (Q1 + Q9) primarily involved acute immune activation components (e.g., antimicrobial peptides, pattern recognition receptors), showing corresponding increases at both mRNA and protein levels. Specifically, Q1 (consistent up-regulation) focused on Toll/Imd signaling pathway components and complement-like proteins, indicating transcription-driven immune pre-activation. Post-transcriptional regulation (Q2 + Q8) emphasized translational control as a primary regulatory mechanism. Q2 (protein up-regulation only) was enriched in metabolic enzymes and chaperones, suggesting stress-induced protein stabilization or enhanced translation efficiency independent of transcriptional changes. Q7 (mRNA up-regulation, protein down-regulation) prominently included secreted immune effectors, while Q3 (mRNA down-regulation, protein up-regulation) contained stress response proteins, likely reflecting compensatory protein stabilization during transcriptional suppression.

Comparison of ARSEF2860 and ZY027 treatments (Figure 6B) showed markedly improved transcriptome–proteome consistency, with tighter clustering along the diagonal and reduced dispersion. Q1 enrichment shifted toward sustained immune maintenance rather than acute activation, reflected by moderate changes in both mRNA and protein abundances.

Joint KEGG enrichment analysis of consistently altered features (Figure 6C) identified oxidative phosphorylation as the most significantly enriched pathway, despite down-regulation at both omics levels. This finding indicated mitochondrial dysfunction as a critical feature of the ZY027 pathogenic mechanism, involving coordinated suppression of respiratory chain components at transcriptional and translational levels. Additionally, enrichment of ubiquinone and other terpenoid-quinone biosynthesis, ECM–receptor interaction, and metabolism of xenobiotics by cytochrome P450 suggested that ZY027 infection significantly disrupted energy metabolism and triggered host detoxification responses.

In comparing the two fungal strains with distinct virulence (Figure 6D), pathways related to carbohydrate metabolism dominated, including pentose phosphate pathway, fructose and mannose metabolism, glycolysis/gluconeogenesis, and carbon metabolism.

## 4. Discussion

This study provides an initial multi-omics characterization of host hemolymph responses during *B. batryticatus* formation at a single infection time point. *B. batryticatus* produced by different *B. bassiana* strains exhibited significant biomass differences (wet and dry weights). These findings indicate an association between fungal strain characteristics and host molecular responses in hemolymph, as well as production yield. Multi-omics analyses demonstrated that fungal strain characteristics profoundly reshaped the molecular expression landscape of silkworm hemolymph. Specifically, ZY027 infection induced strong immune activation, enhanced ribosome biogenesis, and pronounced endoplasmic reticulum stress responses, whereas ARSEF2860 displayed an “immune–metabolic optimization” pattern. These strains showed distinct regulatory effects on key pathways, including the Toll/Imd signaling pathway, energy metabolism, and detoxification responses, suggesting that *B. batryticatus* formation involves fungus–silkworm molecular interactions in hemolymph.

*Bombyx mori* is a well-established model organism in biological and genetic research. It offers comprehensive genomic resources, experimental manipulability, and ease of cultivation, making it ideal for in-depth studies of host–pathogen interactions [25]. Transcriptomic analyses revealed that at 3 days post-infection with *B. bassiana*, silkworm hemolymph underwent extensive and stage-specific gene expression reprogramming. This response did not simply reflect apoptotic activation, but instead represented a highly coordinated and targeted BP. Multiple innate immune pathways were robustly activated, including the *Toll* and *Imd* signaling pathways, *Wnt* signaling pathway, amino sugar and nucleotide sugar metabolism, and aromatic amino acid catabolism. These findings indicate that the host initiates a systematic defense recognition and mobilization program during the early phase of infection.

At the transcriptome level, infection with the ZY027 strain triggered significant ribosome biogenesis (rRNA processing, ribosome assembly) and endoplasmic reticulum stress responses, reflecting anticipated resource allocation for protein synthesis. However, these processes showed inconsistent or diminished patterns at the proteome level, suggesting translational bottlenecks or post-transcriptional regulation. The Toll/Imd pathway consistently enriched at both transcriptomic and proteomic levels across both treatment comparisons, acting as a central hub for host–microbe interactions. However, ARSEF2860 treatment reduced the amplitude of immune pathway enrichment, instead expanding the diversity of metabolic pathways. This pattern, which we tentatively describe as “immune–metabolic modulation,” suggests a relatively attenuated immune response accompanied by metabolic pathway adjustments. However, we caution that this interpretation is based on hemolymph data at a single time point and requires validation across multiple tissues and infection stages. From an ecological and pathophysiological perspective, this optimization may reflect a more “balanced” host–pathogen interaction strategy, where ARSEF2860 avoids triggering catastrophic host immune collapse and massive detoxification responses, thereby allowing more sustained and “milder” nutrient acquisition from host tissues. This “milder” metabolic exploitation strategy may have direct consequences for *B. batryticatus* product quality.

The number of differentially expressed genes at the transcriptional level far exceeded differentially expressed proteins, particularly in the ARSEF2860 vs. ZY027 comparison. This result implies that the protein synthesis capacity of key host tissues may be significantly impaired during late infection stages. This “transcript-to-protein uncoupling” at host defense transcriptional and translational levels, followed by subsequent collapse, likely represents an essential mechanism leading to the ultimate failure of immune defenses in later stages.

By integrating transcriptomic and proteomic data, this study successfully identified key functional modules operating at the core of fungus–silkworm interactions. Comparison of healthy silkworms and ZY027-infected silkworms revealed extensive metabolic reprogramming, particularly evident in changes to energy production systems (oxidative phosphorylation) and defense systems (P450 detoxification enzymes). The comparison between ARSEF2860 and ZY027 strains showed that differences in pathogenicity mainly involved the regulation of central carbon metabolism, especially in the pentose phosphate and glycolysis pathways. These differences likely reflect distinct strategies by the two strains in utilizing host nutritional resources. ARSEF2860 and ZY027 may modulate host carbohydrate metabolism through separate mechanisms, thereby affecting energy availability and biosynthetic precursor synthesis and resulting in different pathogenic outcomes. Therefore, silkworm rigidification involves far more than simple fungal colonization of dead insect bodies. Our multi-omics evidence clearly demonstrates that *B. batryticatus* production is a direct, systemic manifestation and specific endpoint of precise, highly dynamic, and hierarchical molecular interactions between silkworm larvae and invading *B. bassiana*. Therefore, *B. batryticatus* represents a novel “product” shaped within the dead host serving as a bioreactor, driven predominantly by fungal activity.

Studies have revealed significant differences in morphological indicators (e.g., powder frost adhesion, cross-sectional color, hardness) and biochemical indicators (e.g., metabolite content) between white stiff silkworm and green stiff silkworm [26]. Importantly, host hemolymph transcriptomic and proteomic responses induced by different fungal strains also exhibit high specificity [21,22]. While proteomic data revealed activation of cytochrome P450 detoxification enzymes and pathway enrichment for xenobiotic metabolism by cytochrome P450 in ZY027-infected silkworms, we acknowledge that direct metabolomic data are currently lacking. The inferred link between ZY027 infection and specific secondary metabolite production (e.g., beauvericin) is based on pathway enrichment and established literature [27,28], but requires validation through targeted metabolomics and chemical profiling. Specific host gene expression and protein responses triggered by *B. bassiana* influence not only the rate and quality of physiological decline during infection but also directly shape the composition of host-derived components in *B. batryticatus* (e.g., water-soluble proteins, free amino acids). These host-derived substances have potential or confirmed biological activities [29].

Apart from *B. bassiana*, other entomopathogenic fungi capable of infecting silkworms include *Metarhizium* spp., which form “green muscardine” upon infection. Additionally, Cordyceps-related fungi such as *Isaria* spp. [30] and *Paecilomyces* spp. [31] have been confirmed to infect specific Lepidopteran larvae, forming “fungus–insect” complexes. These fungi produce compounds with significant activity on the nervous and cardiovascular systems, such as trichosanthin [32] and cordycepin [33].

Utilizing multi-omics profiling, the identification and selection of *B. bassiana* strains possessing “high-quality *B. batryticatus*-producing traits” has become essential for ensuring product quality [34]. Desired traits include optimal infection dynamics, abundant secondary metabolites, and favorable mycelial growth characteristics. Therefore, standardized high-quality production of *B. batryticatus* depends heavily on rigorous screening and management of clearly defined fungal strains. Extensive isolation, purification, and preservation of various entomopathogenic fungi pathogenic to Lepidopteran insects (e.g., mulberry pyralid, cotton bollworm), emphasizing their biocompatibility and virulence toward silkworm larvae, are both valuable and necessary. Notably, each of these pathways was represented by only one DEP (fructose-bisphosphate aldolase, FBAL), resulting in high rich factors but limited biological support for broad pathway-level remodeling. The same protein was mapped to multiple related carbohydrate metabolism pathways due to its enzymatic function, which inflated the rich factor values. Therefore, we interpret these enrichments cautiously as indicating differential abundance of a single core metabolic enzyme rather than comprehensive pathway reprogramming.

This study primarily focused on hemolymph as a research target, but fungus–silkworm interactions are systemic. Fungi infect not only the hemocoel but also dynamically interact within muscles, fat bodies, silk glands, midgut, and even the central nervous system [21,35]. Future research should employ spatial transcriptomic/proteomic technologies (spatial omics) to examine interaction patterns and coordination across different tissues. Advanced methods such as continuous monitoring or single-cell sequencing may reveal precise regulatory switches or dynamic balances of key molecular nodes. Additionally, quality assessment should incorporate both target medicinal components (e.g., key fungus-derived secondary metabolites, host-derived active peptides) and stability indicators (consistency of morphology, physical properties, and chemical composition between batches).

In conclusion, this study comprehensively analyzed, for the first time, the highly dynamic and complex molecular mechanisms underlying *B. batryticatus* formation in silkworms infected by *B. bassiana* using integrated transcriptomic and proteomic approaches. Based on systematic molecular evidence, this study proposes three core concepts: (1) *B. batryticatus* is fundamentally a specialized fungus–silkworm interaction product; (2) fungal characteristics predominantly determine the intrinsic quality of *B. batryticatus*; (3) moving beyond a single *B. batryticatus* production approach by fully exploiting diverse entomopathogenic fungal resources with specific medicinal properties and potential for strain engineering represents a crucial innovative strategy for enhancing its medicinal value. This research deepens understanding of *B. batryticatus* as a distinctive traditional Chinese medicinal resource and opens new opportunities for its high-quality traditional and modern integrated development, including the discovery of novel medicinal resources and intelligent manufacturing practices.

## 5. Conclusions

This study provides an initial multi-omics analysis of host hemolymph responses during *B. batryticatus* formation, revealing strain-dependent molecular reprogramming patterns associated with fungal infection. Our findings demonstrate that two *B. bassiana* strains with different virulence induce distinct host transcriptomic and proteomic responses in hemolymph, which are associated with significant biomass differences in the final product. These results suggest that fungal strain selection is an important factor influencing *B. batryticatus* production yield and host molecular phenotypes. However, we emphasize that (1) these conclusions are based on hemolymph at a single infection time point and do not represent the full multi-tissue, multi-stage process of *B. batryticatus* formation; (2) biomass measurements alone are insufficient proxies for medicinal quality; and (3) validation by RT-qPCR, metabolomic profiling, and bioactivity assays is needed to establish quality-related conclusions. This work provides a foundation for future systematic investigations of fungus–silkworm interactions and their implications for *B. batryticatus* production and quality standardization.

## Data Availability

The data presented in this study are available in manuscript. The original data on proteomics have been uploaded to the ProteomeXchange Database (http://proteomecentral.proteomexchange.org/cgi/GetDataset?ID=PXD074859 (accessed on 24 February 2026)). The original data on transcriptomics have been uploaded to OMIX, China National Center for Bioinformation/Beijing Institute of Genomics, Chinese Academy of Sciences (https://ngdc.cncb.ac.cn/gsa/browse/CRA039075 (accessed on 23 February 2026)).
